# Perceived characteristics of the environment associated with active travel: development and testing of a new scale

**DOI:** 10.1186/1479-5868-5-32

**Published:** 2008-05-30

**Authors:** David Ogilvie, Richard Mitchell, Nanette Mutrie, Mark Petticrew, Stephen Platt

**Affiliations:** 1 Medical Research Council Social and Public Health Sciences Unit, Glasgow, UK; 2 Section of Public Health and Health Policy, University of Glasgow, Glasgow, UK; 3 Department of Sport, Culture and the Arts, University of Strathclyde, Glasgow, UK; 4 London School of Hygiene and Tropical Medicine, London, UK; 5 Research Unit in Health, Behaviour and Change, University of Edinburgh, Edinburgh, UK; 6 Medical Research Council Epidemiology Unit, Cambridge, UK

## Abstract

**Background:**

Environmental characteristics may be associated with patterns of physical activity. However, the development of instruments to measure perceived characteristics of the local environment is still at a comparatively early stage, and published instruments are not necessarily suitable for application in all settings. We therefore developed and established the test-retest reliability of a new scale for use in a study of the correlates of active travel and overall physical activity in deprived urban neighbourhoods in Glasgow, Scotland.

**Methods:**

We developed and piloted a 14-item scale based on seven constructs identified from the literature (aesthetics, green space, access to amenities, convenience of routes, traffic, road safety and personal safety). We administered the scale to all participants in a random postal survey (n = 1322) and readministered the scale to a subset of original respondents (n = 125) six months later. We used principal components analysis and Varimax rotation to identify three principal components (factors) and derived summary scores for subscales based on these factors. We examined the internal consistency of these subscales using Cronbach's alpha and examined the test-retest reliability of the individual items, the subscale summary scores and an overall summary neighbourhood score using a combination of correlation coefficients and Cohen's kappa with and without weighting.

**Results:**

Public transport and proximity to shops were the items most likely to be rated positively, whereas traffic volume, traffic noise and road safety for cyclists were most likely to be rated negatively. Three principal components – 'safe and pleasant surroundings', 'low traffic' and 'convenience for walking' – together explained 45% of the total variance. The test-retest reliability of individual items was comparable with that of items in other published scales (intraclass correlation coefficients (ICCs) 0.34–0.70; weighted Cohen's kappa 0.24–0.59). The overall summary neighbourhood score had acceptable internal consistency (Cronbach's alpha 0.72) and test-retest reliability (ICC 0.73).

**Conclusion:**

This new scale contributes to the development of a growing set of tools for investigating the role of perceived environmental characteristics in explaining or mediating patterns of active travel and physical activity.

## Background

A growing body of evidence suggests that certain characteristics of the physical environment may be associated with patterns of physical activity in general or with particular types of physical activity such as walking or cycling as modes of transport. [1-7] Physical activity has been found to be associated with both 'objective' characteristics of the environment – such as those ascertained using observations made by trained researchers, or using spatially referenced census and transport infrastructure data held and analysed in a geographical information system (GIS) – and with characteristics of the environment as perceived and reported by study participants. However, the cumulated evidence about the strength and direction of associations with objective or perceived environmental characteristics is far from conclusive and is dominated by cross-sectional studies, mostly conducted in North America and Australia. [4,6,8,9] Hypotheses about putative environmental correlates of physical activity therefore need to be tested in a wider range of settings and in longitudinal studies capable of establishing a temporal relationship between exposure to particular environmental characteristics and consequent changes in behaviour.

We established a longitudinal study to examine changes in travel behaviour and physical activity associated with the opening of a new urban section of the M74 motorway (freeway) currently under construction in Glasgow, Scotland. [10] In the cross-sectional (baseline) phase of the study, we wished to test the hypotheses that levels of 'active travel' (walking and cycling for transport) and overall physical activity vary with demographic and socioeconomic characteristics, but not necessarily in the same way, and that these relationships may be partly explained by the perceived characteristics of the local environment in which people live – as well as by their objectively-assessed proximity to motorway and major road infrastructure. We also wished to be able to test, in the future, the hypothesis that this major modification to the urban built environment will be associated with changes in active travel which may be partly explained or mediated by changes in the perceived characteristics of the local environment.

The development of valid and reliable instruments to measure perceived characteristics of the local environment is still at a comparatively early stage. [3,11,12] For our purposes, it seemed important that the chosen instrument should satisfy three criteria. First, it should measure constructs shown (at least in some studies) to be related to physical activity in general or walking and cycling in particular and that could reasonably be expected to change as a result of the intervention. Second, it should have face validity in the local context. Third, it should be suitable for completion as part of a postal survey.

From a number of recent reviews, we identified seven constructs that met the first criterion: aesthetics, green space, access to amenities, convenience of routes, traffic, road safety and personal safety. [1-7] However, instruments for measuring these constructs which have been validated and published typically also include items on non-modifiable characteristics which could not conceivably change as a result of the intervention, thereby failing our first criterion, or refer to irrelevant characteristics or use inappropriate language for the urban setting in Glasgow, thereby failing our second criterion. To give two examples, the scale published by Humpel and colleagues includes one item on hilliness and four items on weather (both of which could be regarded as non-modifiable characteristics), and asks whether there is a lake or beach within walking distance (an irrelevant question in our study areas), [12] and the Neighbourhood Environment Walkability Scale (NEWS) is written in American English and asks about the presence of sidewalks, which are ubiquitous in Glasgow. [13] NEWS also illustrates the difficulties posed by our third criterion: in its original version it contains 83 items over seven pages, which we judged too long for inclusion in a 'cold-call' postal survey of the general population which also had to include instruments for measuring travel behaviour, physical activity, and health and wellbeing.

We therefore designed, piloted and established the test-retest reliability of our own instrument. In this paper, we report the development of this 'neighbourhood scale' in the context of an observational intervention study in a deprived urban population in the west of Scotland. This paper therefore underpins our companion paper, in which we report the result of our analyses of the personal and environmental correlates of active travel and physical activity (Ogilvie et al, submitted for publication), and contributes to the development of a growing set of tools which may be useful for researchers working in other settings.

## Methods

### Measures of perceived characteristics of the local environment

We devised a scale to assess perceived characteristics of the local environment based on the constructs identified from the literature. We asked respondents to rate their agreement with 14 items on a five-point scale (from 'strongly agree' to 'strongly disagree'). The scale comprised pairs of items reflecting each of the seven constructs of interest. We piloted these items in a small test-retest study of academic staff and students and members of the general population (n = 23, test-retest interval 7–18 days) and found them all to have acceptable test-retest correlation (Spearman's rank correlation coefficient (*r*_s_) ranging from 0.55 to 0.89) except for one item, 'There are no shops within walking distance' (*r*_s _= 0.21), which we subsequently reworded to 'The nearest shops are too far to walk to'. In the final scale, the pair of items reflecting each construct comprised one positively-worded item and one negatively-worded item, and the 14 items were presented to respondents in such an order that positively worded items alternated with negatively worded items (Table 1). For comparison, we also asked respondents to indicate on a seven-point rating scale 'Which face shows best how you feel about living in your local area?' (Additional file 1, page 6).

**Table 1:** Items in neighbourhood scale

Construct	Item	Order*
Aesthetics	It is pleasant to walk	1
	The surroundings are unattractive	14
Green space	There is a park within walking distance	3
	There is little green space	8
Access to amenities	There is convenient public transport	5
	The nearest shops are too far to walk to	10
Convenience of routes	There are convenient routes for cycling	7
	There are no convenient routes for walking	12
Traffic	There is little traffic	11
	There is a lot of traffic noise	2
Road safety	It is safe to cross the road	13
	The roads are dangerous for cyclists	4
Personal safety	It is safe to walk after dark	9
	People are likely to be attacked	6

* Order in which items were presented to respondents

### Sampling and administration of survey

#### Main survey

The methods of sampling and data collection are described more fully in the accompanying paper (Ogilvie et al, submitted for publication). Briefly, we delineated three matching study areas on the basis of spatial and aggregate socioeconomic characteristics, identified a random sample of households in these areas using the Royal Mail Postcode Address File (PAF), and surveyed adults living in these households using a postal questionnaire which included items on demographic and socioeconomic characteristics, health and wellbeing, perceptions of the local environment, travel behaviour and physical activity (Additional file 1). The 'neighbourhood scale' which we developed to assess perceptions of the local environment is the subject of this paper.

#### Test-retest reliability study

In order to establish the test-retest reliability of the neighbourhood scale, we sent a retest questionnaire to a stratified random sample of the original respondents on 16 March 2006 (approximately six months after the original survey). The sampling frame for the retest survey comprised all respondents to the original survey who had replied within one month, given their consent for follow-up and supplied a full set of responses to the neighbourhood scale; who did not live in one of a small number of streets selected for a separate qualitative interview study; and who did not live within 500 metres of any of four sites where we had identified a significant change to the local area occurring around or since the time of the original survey (major housing development or the opening of a new railway station). We partitioned this sampling frame by sex and tertile of age and selected a stratified random sample of 200 participants to receive the retest survey, which consisted of the neighbourhood scale and a question to confirm that respondents were still living at the same address. Respondents were offered the modest incentive of a £5 (€7; US$10) gift voucher in exchange for a completed retest questionnaire. We compared selected characteristics of the achieved retest sample with those of the remainder of the achieved sample for the main survey using the chi-squared test.

### Principal components analysis

We used principal components analysis to explore the underlying structure, and reduce the complexity, of the data obtained using the neighbourhood scale in the main survey. The principle of factor analysis is that by examining correlations between a set of variables it may be possible to identify a smaller number of underlying factors (components) which explain much of the variance in the original variables – either to confirm factors believed *a priori *to be significant (confirmatory factor analysis), or (as in this case) in a situation where no such *a priori *belief is declared (exploratory factor analysis). Statistical packages such as SPSS offer numerous alternative methods for exploratory factor analysis. In the absence of any clear consensus in the literature as to which is most appropriate for the analysis of social data, we considered two alternative methods: maximum-likelihood exploratory factor analysis, which some regard as the 'truest' method in statistical terms, [14] and principal components analysis (PCA), which has more often been used in practice in this field. Preliminary maximum-likelihood exploratory factor analysis suggested that at least eight extracted factors would be required to fit the data adequately (data not shown). We did not consider this a useful reduction in complexity from the 14 original items in the scale. We therefore proceeded with PCA using the SPSS function *Factor Analysis: Extraction: Principal Components*. To begin with, we did not specify the number of components to be extracted. We confirmed the suitability of the data for PCA using the Kaiser-Meyer-Olkin measure of sampling adequacy (a measure of the degree of common variance between the variables) and Bartlett's test of sphericity (a test of the null hypothesis that the variables are completely uncorrelated). We then considered the eigenvalues for the extracted components against standard criteria which can be used to select the 'important' components (those above the change in slope of the scree plot, or those with eigenvalues greater than one). Adapting the method used by Humpel and colleagues in their study of percevied environmental characteristics related to walking, [15] we then repeated the analysis applying Varimax rotation to simplify the interpretation, identified the correlation coefficients (loading factors) for each scale item on the components extracted, and considered whether the group of items most strongly correlated with each extracted component could be interpreted and labelled using a single overarching construct. Having selected the three most appropriate components (see results), we then calculated summary scores for each of three neighbourhood subscales, defined as the sum of the scores for the individual items most strongly correlated with each of the three principal components. We then described the distribution of summary scores for each subscale and calculated Cronbach's α (a measure of internal consistency) and measures of test-rest reliability for each subscale and for the neighbourhood scale as a whole.

### Test-retest reliability analysis

Different authors have used different measures to report the test-retest reliability of similar items in the published literature. In the interests of comparability, we therefore cross-tabulated the test and retest responses to each item on the neighbourhood scale and calculated several alternative measures of reliability: the percentage exact agreement, the Pearson, Spearman and intraclass correlation coefficients, the chance-corrected agreement (Cohen's κ), and the chance-corrected agreement after collapsing each item from a five-point scale to a three-point scale – a weighted version of Cohen's κ to take account of the fact that, for example, a change in response from negative to zero, or from negative to positive, could be considered more significant than a change from -2 to -1.

## Results

### Characteristics of study participants

The characteristics of the study participants are described more fully in the accompanying paper (Ogilvie et al, submitted for publication). Briefly, the main sample comprised 1322 adults aged between 16 and 89 years (median age 48 years), of whom 61% were women; 47% were in full- or part-time employment, and 52% lived in owner-occupied accommodation; 48% had no household access to a car or van, and only 21% had access to a bicycle; 25% reported difficulty walking for a quarter of a mile, 39% reported a long-term health problem or disability, and 50% were overweight.

Most respondents were long-term residents of their local area (median duration of residence 14 years). As expected, duration of residence was correlated with age (*r*_s _= 0.56).

660 respondents met the criteria for inclusion in the sampling frame for the test-retest reliability study, of whom a random sample of 200 stratified by sex and tertile of age (cut-points 38 and 54 years) received the retest questionnaire. Of these, 125 (63%) returned valid retest responses. The achieved retest sample had a balanced representation of men (n = 63) and women (n = 62) and an age distribution similar to that of the initial sample (median age 47 years). There were no significant differences between retest respondents and the remainder of the achieved sample for the main survey in the proportions who lived in owner-occupied households (57% vs 51%, P = 0.22), had access to a car (56% vs 52%, P = 0.35), reported active travel (29% vs 28%, P = 0.93) or reported sufficient physical activity (43% vs 37%, P = 0.28).

### Perceived characteristics of the local environment

**Figure 1:**
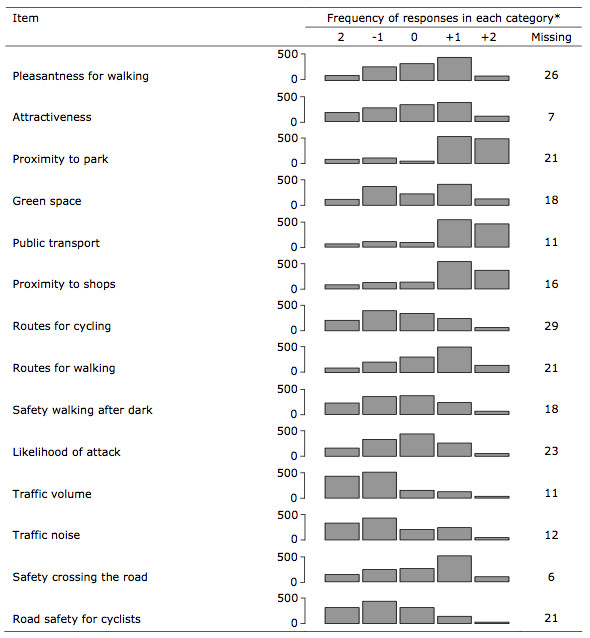
**Responses to individual items on neighbourhood scale**. n = 1322. * Items recoded such that +2 is the most favourable and -2 the least favourable response category for each item.

The distributions of responses in the main survey to each of the 14 items on the neighbourhood scale are displayed with the responses to the negatively-worded items recoded so that for each item a value of +2 represents the most favourable response ('strongly agree' or 'strongly disagree', as appropriate), a value of -2 represents the least favourable response, and a value of zero represents a neutral response ('neither agree nor disagree') (Figure 1). The items which most often elicited the most favourable (+2) response were those on public transport and proximity to shops; the items which most often elicited the least favourable (-2) response were those on traffic volume, traffic noise and road safety for cyclists. There was little evidence of collinearity between the items: no pair of items had a correlation coefficient greater than 0.5, and most pairwise correlation coefficients were less than 0.2 (Table 2).

**Table 2:** Pearson correlation matrix for neighbourhood scale items

	Pleasantness for walking	Attractiveness	Proximity to park	Green space	Public transport	Proximity to shops	Routes for cycling	Routes for walking	Safety walking after dark	Likelihood of attack	Traffic volume	Traffic noise	Safety crossing the road
Attractiveness	0.48												
Proximity to park	0.23	0.16											
Green space	0.18	0.26	0.26										
Public transport	0.19	0.10	0.16	0.01									
Proximity to shops	0.06	0.09	0.19	0.16	0.16								
Routes for cycling	0.24	0.15	0.14	0.09	0.04	-0.06							
Routes for walking	0.29	0.33	0.23	0.29	0.13	0.29	0.17						
Safety walking after dark	0.37	0.27	0.18	0.10	0.15	0.05	0.22	0.13					
Likelihood of attack	0.33	0.38	0.06	0.21	0.04	0.06	0.15	0.24	0.42				
Traffic volume	0.18	0.08	-0.05	0.06	-0.14	-0.16	0.19	-0.03	0.16	0.13			
Traffic noise	0.11	0.18	-0.02	0.13	-0.17	-0.03	0.08	0.08	0.08	0.20	0.50		
Safety crossing the road	0.21	0.15	0.19	0.11	0.12	0.03	0.18	0.13	0.25	0.18	0.29	0.22	
Road safety for cyclists	0.14	0.18	-0.02	0.12	-0.08	-0.01	0.22	0.15	0.12	0.27	0.28	0.40	0.27

n = 125

### Summary scores

The average summary neighbourhood score (the sum of these 14 individual items) was zero (mean score 0.2, standard deviation 7.2; median score 0.0, interquartile range 10.0) and the internal consistency of this summary score was satisfactory (Cronbach's α = 0.72). [16] When asked 'Which face shows best how you feel about living in your local area?', 846 respondents (64%) selected one of the three smiling faces, i.e. a response category more favourable than the midpoint of the seven-point rating scale. There was a significant association between the tertile of summary neighbourhood score (cut-points ≤-3 and ≥4) and the response to the single item 'How do you feel about living in your local area?' (test for linear trend: χ^2 ^= 227.12, df = 1; p < 0.001).

### Principal components analysis and development of subscales

**Figure 2:**
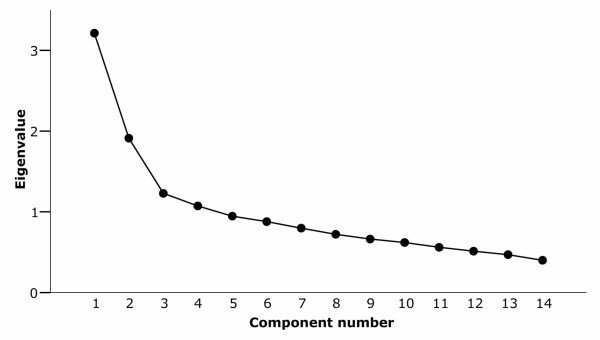
Scree plot of eigenvalues associated with principal components.

Initial PCA extracted 14 components and returned satisfactory values for tests to confirm that the data were suitable for PCA (Kaiser-Meyer-Olkin measure of sampling adequacy 0.76; Bartlett's test of sphericity: χ^2 ^= 3222.14, df = 91; p < 0.001). The scree plot showed a flattening of the eigenvalue curve after the first three factors, which together explained 45% of the variance; on the other hand, the first four factors had an eigenvalue >1 and together explained 53% of the variance (Figure 2). We therefore repeated the analysis, specifying first a three- and then a four-factor solution and applying Varimax rotation (Table 3). The four items which 'loaded significantly' onto the first factor of the four-factor solution could more easily be interpreted and labelled using a single overarching construct ('safe and pleasant surroundings') than the six items which 'loaded significantly' onto the first factor of the three-factor solution. In addition, there was no cross-loading of items between factors in the four-factor solution, whereas in the three-factor solution, attractiveness 'loaded significantly' onto both the first and third factors. We therefore defined the three principal components (factors) as those most strongly correlated with the first three factors of the four-factor solution, labelling these factors as factor 1, 'safe and pleasant surroundings'; factor 2, 'low traffic'; and factor 3, 'convenience for walking' (Table 4). There was little evidence of collinearity between the summary scores for these subscales (pairwise correlation coefficients -0.03, 0.17 and 0.34). The internal consistency of the subscale based on factor 1 ('safe and pleasant surroundings') was satisfactory (Cronbach's α = 0.70), whereas that of the subscales based on factors 2 and 3 was lower (Cronbach's α = 0.58 and 0.55 respectively).

**Table 3:** Varimax-rotated component matrices

	Three-factor solution			Four-factor solution			
	
	Component number			Component number			
	
Item	1	2	3	1	2	3	4
Pleasantness for walking	0.69			0.67			
Attractiveness	0.49		0.42	0.70			
Proximity to park			0.45			0.49	0.53
Green space			0.64			0.62	
Public transport		-0.50			-0.43		0.52
Proximity to shops			0.66			0.68	
Routes for cycling	0.49						0.46
Routes for walking			0.70			0.65	
Safety walking after dark	0.73			0.63			
Likelihood of attack	0.54			0.74			
Traffic volume		0.67			0.72		
Traffic noise		0.79			0.80		
Safety crossing the road	0.49						0.66
Road safety for cyclists		0.64			0.65		

Correlation coefficients (loading factors) with absolute values <0.4 omitted in the interests of clarity

**Table 4:** Neighbourhood factors identified using principal components analysis

Factor	Items contributing to factor	Direction of correlation	Label for factor
1	Pleasantness for walking	Positive	Safe and pleasant surroundings
	Attractiveness	Positive	
	Safety walking after dark	Positive	
	Likelihood of attack	Positive	

2	Public transport	Negative	Low traffic
	Traffic volume	Positive	
	Traffic noise	Positive	
	Road safety for cyclists	Positive	

3	Proximity to park	Positive	Convenience for walking
	Green space	Positive	
	Proximity to shops	Positive	
	Routes for walking	Positive	

### Test-retest reliability

The test-retest characteristics of each item on the neighbourhood scale and of the summary scores are summarised in Table 5.

**Table 5:** Test-retest relationships for neighbourhood scale items and summary scores

	Measure of test-retest agreement					
	
		Correlation coefficient			Cohen's κ	
		
Item	Exact agreement*	Pearson	Spearman	Intraclass	Original categories	Collapsed categories
**Individual item**						
Pleasantness for walking	62 (49.6)	0.64	0.63	0.64	0.32	0.44
Attractiveness	67 (53.6)	0.58	0.60	0.58	0.38	0.47
Proximity to park	75 (60.0)	0.54	0.54	0.54	0.38	0.47
Green space	50 (40.0)	0.36	0.38	0.36	0.18	0.24
Public transport	83 (66.4)	0.70	0.66	0.70	0.50	0.59
Proximity to shops	82 (65.6)	0.51	0.55	0.50	0.50	0.53
Routes for cycling	56 (44.8)	0.53	0.54	0.52	0.26	0.39
Routes for walking	66 (52.8)	0.33	0.40	0.34	0.34	0.36
Safety walking after dark	61 (48.8)	0.42	0.46	0.42	0.38	0.41
Likelihood of attack	62 (49.6)	0.68	0.66	0.67	0.32	0.42
Traffic volume	73 (58.4)	0.47	0.56	0.48	0.40	0.35
Traffic noise	68 (54.4)	0.63	0.64	0.62	0.38	0.53
Safety crossing the road	57 (45.6)	0.52	0.47	0.52	0.20	0.32
Road safety for cyclists	59 (47.2)	0.45	0.49	0.45	0.28	0.37
**Summary score**^†^						
Factor 1 subscale	--	0.75	0.72	0.75	--	--
Factor 2 subscale	--	0.75	0.76	0.75	--	--
Factor 3 subscale	--	0.57	0.59	0.57	--	--
Overall score	--	0.73	0.73	0.73	--	--

n = 125. * Count (%). †Exact agreement and Cohen's κ not applicable to continuous summary variables

The proportion of respondents who gave exactly the same response to a particular item at test and retest ranged from 40% (for 'There is little green space') to 66% (for 'There is convenient public transport'). The constructs (pairs of items) most likely to elicit exactly the same response at retest were those concerned with access to amenities and with traffic; the constructs least likely to elicit a consistent response were those concerned with road safety and personal safety. Test-retest correlation coefficients for each item ranged from 0.33 to 0.70 (Pearson), from 0.38 to 0.66 (Spearman), or from 0.34 to 0.70 (intraclass). The choice of method made little difference to the estimates of the coefficients; irrespective of method, the constructs (pairs of items) with the strongest test-retest correlations were those concerned with aesthetics and access to amenities, whereas the constructs with the weakest test-retest correlations were those concerned with green space and convenience of routes. The unweighted value of Cohen's κ for the chance-adjusted test-retest agreement for each item ranged from 0.18 to 0.50. Thirteen items had a value of κ≥0.20 ('fair' agreement), while three had a value of κ≥0.40 ('moderate' agreement): these were the two items concerned with access to amenities (both κ = 0.50) and 'There is little traffic' (κ = 0.40). After collapsing the five response categories into three categories (positive, neutral and negative), the recalculated values of Cohen's κ ranged from 0.24 to 0.59, with 8 of the 14 items having a recalculated value of κ≥0.40 ('moderate' agreement).

The test-retest correlation coefficient for the summary neighbourhood score was 0.73, irrespective of the choice of method.

## Discussion

### Performance of scale

The neighbourhood scale devised for this study appeared to perform adequately for our purposes, although it should be noted that we have only assessed its performance in one particular urban context. Further studies would be required to confirm its capacity to discriminate between a wider variety of types of neighbourhood and to determine whether the reliability of the items in the scale is generalisable between settings.

First, the correlation matrix showed little evidence of collinearity between the items, even within pairs of items. This suggests that, as intended, the different items were measuring different aspects of respondents' perceptions of their surroundings.

Second, although the test-retest reliability of the items was not as high as might appear desirable according to the criteria typically specified in statistical textbooks, the results of our reliability analyses were broadly comparable with those achieved in other studies of similar instruments which used four- or five-point rating scales – especially taking into account the long test-retest interval in this study (six months). We chose to analyse test-retest reliability using a variety of measures in order to maximise comparability with other studies, which have tended to use the intraclass correlation coefficient (ICC) as a measure of reliability. For example, in their assessment of a new 17-item module to assess perceptions of the local environment as an adjunct to the International Physical Activity Questionnaire (IPAQ) in a Swedish population, Alexander and colleagues reported test-retest ICCs for individual items ranging from 0.36 to 0.98; however, their test-retest interval was only one week, and the only two items with an ICC of greater than 0.9 involved asking questions which appeared considerably more concrete and objective than those addressed in our scale ('What is the main type of housing in your neighborhood?' and 'How many motor vehicles in working order are there in your household?'). [17] Similarly, in a study of three questionnaires developed in the United States (the South Carolina and St. Louis instruments and the San Diego instrument, the latter now being known as NEWS) using a test-retest interval of one to three weeks, Brownson and colleagues reported ICCs for individual items comparable with those used in our scale ranging from 0.39 to 0.87, from 0.36 to 0.80 and from 0.18 to 0.78 in the three sites respectively. [11]

Although the precise ranking of the reliability of the individual items varied according to which of these metrics was used (e.g. using ICC compared with Cohen's κ), across all metrics the item which stood out as being the most reliable was that on access to public transport. This may reflect a greater degree of certainty (and therefore relative lack of intra-subject variation) in respondents' assessment of their access to public transport, which might be interpreted in a more concrete way than their assessment of more subjective characteristics such as the attractiveness of their surroundings.

Third, the summary score obtained by summing the responses to the individual items in the scale was approximately symmetrically distributed about a mean of zero, had acceptable test-retest reliability, and exhibited a highly significant association with the alternative single-item rating scale 'How do you feel about living in your local area', suggesting acceptable concurrent validity in the terms within which this could be established in this context.

Fourth, principal components analysis suggested three latent factors capable of being interpreted and labelled, and summary scores calculated by summing the scores for the items most significantly associated with each factor had acceptable internal consistency and test-retest reliability and did not exhibit significant collinearity.

### Interpretation of findings

The contribution of the neighbourhood scale to understanding the correlates of active travel and physical activity in our study population is discussed in the accompanying paper (Ogilvie et al, submitted for publication), which may be briefly summarised as showing that differences in perceptions of the local environment accounted for little additional variance in either active travel or physical activity after personal characteristics were taken into account. However, this more-or-less 'negative result' for environmental correlates overall does not necessarily reflect poorly on the capacity of our neighbourhood scale to measure meaningful constructs.

As shown in Figure 1, the distribution of responses varied markedly between items on the scale. For some items, the peak of the distribution was at the midpoint of the scale (e.g. safety walking after dark and likelihood of attack) whereas for others, the distribution exhibited a clear skew towards positive responses (e.g. proximity to a park, public transport, proximity to shops and safety crossing the road) or negative responses (e.g. traffic volume, traffic noise and road safety for cyclists). In other words, although the average summary score for the scale was close to the midpoint (zero, representing a neutral response), this neutral aggregate opinion of the overall characteristics of the local environment conceals widely divergent aggregate opinions about specific characteristics. This suggests that it is likely to be important to attempt to measure these specific characteristics rather than relying on a simple overall rating of the local environment.

The skewness of the distribution of responses to some items is unsurprising; for example, it should come as no surprise that residents of a comparatively densely-developed conurbation with an extensive bus network should tend to rate traffic volume, traffic noise, proximity to shops and the availability of public transport highly. On the other hand, we found an interesting divergence between a predominantly positive perception of road safety for pedestrians and a predominantly negative perception of road safety for cyclists. This may be a real divergence, possibly related to the low prevalence of access to bicycles in the study population, which could be explored further in a future qualitative study; however, it should be noted that these were the items with the poorest test-retest reliability.

Principal components analysis suggested that the items could be grouped into three more general factors reflecting the safety and pleasantness of the surroundings, the lack of road traffic, and the convenience of routes and destinations for walking – all of which have some face validity as being plausibly associated with active travel in the local area. Despite the fact that none of these general factors were shown to be significantly associated with active travel or physical activity (Ogilvie et al, submitted for publication), our analysis highlights the possibility that different neighbourhoods may 'score highly' on one, but not all, of these apparently desirable characteristics – for example, neighbourhoods with comparatively little traffic may also be perceived as safe and pleasant but may not have local amenities which can be conveniently reached on foot.

## Conclusion

We successfully developed and used a new, short (14-item) neighbourhood scale suitable for assessing residents' perceptions of the local environment in the context of an observational study of the effects of a major modification to the urban built environment and transport infrastructure in a comparatively deprived urban population in the west of Scotland, and showed this scale to have acceptable test-retest reliability compared with that of related scales developed for use in other contexts.

This work therefore contributes to the development of a growing set of tools which may be useful for researchers investigating the role of perceived environmental characteristics, and changes in those characteristics, in explaining or mediating patterns of active travel and physical activity and how these are affected by interventions which change the physical environment.

## Competing interests

This paper is based on material contained in the first author's PhD thesis.

## Authors' contributions

DO had the original idea for the study, designed the study and the survey materials, applied for ethical approval, cleaned and coded the survey data, carried out all the analyses and wrote the paper. MP was DO's PhD supervisor. RM, NM, MP and SP constituted the steering group for the study, contributed to and advised on the design of the study and the interpretation of the emerging findings, contributed to the critical revision of the paper, and approved the final version.

## Supplementary Material

Additional file 1 Survey questionnaire. Click here for file
