# Supplementary material for: Perceived characteristics of the environment associated with active travel: development and testing of a new scale
Source: Int J Behav Nutr Phys Act. 2008 May 30;5:32. doi: 10.1186/1479-5868-5-32 (PMC2416440; doi:10.1186/1479-5868-5-32)
Supplement: Additional file 1 — Survey questionnaire. [file 1479-5868-5-32-S1.pdf]

UNIVERSITY of GLASGOW

MRC Medical Research Council

Traffic and health in Glasgow Questionnaire

CONFIDENTIAL

Who should complete this questionnaire

This questionnaire should be completed by **one adult member of the household** (aged 16 and over) to which the envelope was addressed. Please do **not** pass it on to another household.

If there is more than one adult in the household, please choose the adult who has most recently had their birthday to complete the questionnaire. For example, if one person has a birthday in March and the other has a birthday in May, please choose the person whose birthday is in May.

How to complete the questionnaire

The questionnaire is not long. It should take about 15 minutes to complete.

Please use a blue or black pen.

Some questions ask you to **tick** a box. Please tick the box that applies to you.

Example: Are you male or female? Male ☑ Female ☐

Other questions ask you to **write numbers** in a box.

Example: What is your age? *Write in* [53] years

Don't worry if you make a **mistake** — just cross out the mistake and put in the correct answer.

Example: Do you have access to a bicycle? Yes ☒ No ☑

About you and your household

1 Are you male or female? *Tick one only* Male ☐ Female ☐

2 What is your age? *Write in* ☐ years

3 How far do you have to travel to get to your usual place of work or study?

Tick one only

Do not work or study ☐

Usually work at home or from home ☐

Less than one mile ☐

One mile or more ☐ → *Write in number of miles* ☐

4 Do you have access to a bicycle?

Tick one only Yes ☐ No ☐

5 How many other people live in your household?
We mean people who have your accommodation as their only or main residence, and who either share at least one meal a day with you or share the living accommodation (living room or sitting room) with you.

Write in number
If none, write "0"

Children aged under 5 ☐

Children aged between 5 and 15 ☐

Adults aged 16 and over (do not include yourself) ☐

6 Does your household own or rent its accommodation?

Tick one only

Rents it from the council, Scottish Homes, a housing association, or a charity ☐

Rents it from a private landlord or letting agency ☐

Partly owns it and partly rents it (shared ownership) ☐

Owns it (including buying with a mortgage) ☐

Other ☐

7 **How many cars or vans are owned, or available for use, by members of your household?**
Do not include motorcycles, scooters or mopeds.

Write in number
If none, write "0" ☐

8 **Thinking about the work you do, which of these best describes your situation at present?**
Please answer for yourself, and for your spouse or partner if you have one who lives with you.

	Yourself<br>*Tick one only*	Your spouse/partner<br>*Tick one only*
Doing paid work full time	☐	☐
Doing paid work part time	☐	☐
On a government training scheme	☐	☐
Retired	☐	☐
Full time student	☐	☐
Unemployed	☐	☐
Disabled, invalid or permanently sick	☐	☐
Caring for home and family or dependants	☐	☐
Other	☐	☐
Not living with a spouse or partner		☐

9 **Thinking about the cost of living as it affects you and your household, which of these best describes your situation at present?**

	Tick one only
Find it a strain to get by from week to week	☐
Have to be careful about money	☐
Able to manage without much difficulty	☐
Quite comfortably off	☐

About your health

10 Looking at the faces scale, which face shows best how you feel about your life as it is now?

Tick one only

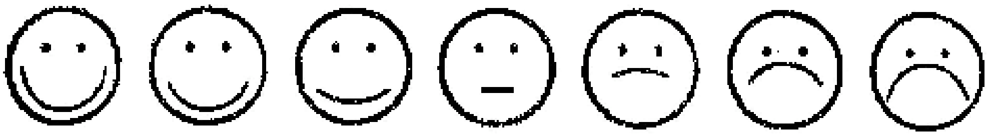

☐ ☐ ☐ ☐ ☐ ☐ ☐

11 Do you have any long-term illness, health problem or disability which limits your daily activities or the work you can do? Include problems which are due to old age.

Tick one only Yes ☐ No ☐

12 Do you have any difficulty walking for a quarter of a mile on the level?

Tick one only Yes ☐ No ☐

13 How tall are you? (with your shoes off)

Write in ☐ ft ☐ in **OR** ☐ cm

14 How much do you weigh? (in light indoor clothes)

Write in ☐ st ☐ lb **OR** ☐ kg

The next section asks for your views about your health. This information will help keep track of how you feel and how well you are able to do your usual activities.

For each of the following questions, please tick the one box that best describes your answer.

15 Overall, how would you rate your health during the PAST FOUR WEEKS?

Excellent	Very good	Good	Fair	Poor	Very poor
☐	☐	☐	☐	☐	☐

16 During the PAST FOUR WEEKS, how much did physical health problems limit your usual physical activities (such as walking or climbing stairs)?

Not at all	Very little	Somewhat	Quite a lot	Could not do physical activities
☐	☐	☐	☐	☐

17 During the PAST FOUR WEEKS, how much difficulty did you have doing your daily work, both at home and away from home, because of your physical health?

None at all	A little bit	Some	Quite a lot	Could not do daily work
☐	☐	☐	☐	☐

18 How much BODILY pain have you had during the PAST FOUR WEEKS?

None	Very mild	Mild	Moderate	Severe	Very severe
☐	☐	☐	☐	☐	☐

19 During the PAST FOUR WEEKS, how much energy did you have?

Very much	Quite a lot	Some	A little	None
☐	☐	☐	☐	☐

20 During the PAST FOUR WEEKS, how much did your physical health or emotional problems limit your usual social activities with family or friends?

Not at all	Very little	Somewhat	Quite a lot	Could not do social activities
☐	☐	☐	☐	☐

21 During the PAST FOUR WEEKS, how much have you been bothered by emotional problems (such as feeling anxious, depressed or irritable)?

Not at all	Slightly	Moderately	Quite a lot	Extremely
☐	☐	☐	☐	☐

22 During the PAST FOUR WEEKS, how much did personal or emotional problems keep you from doing your usual work, school or other daily activities?

Not at all	Very little	Somewhat	Quite a lot	Could not do daily activities
☐	☐	☐	☐	☐

SF-8™ 4-Week Recall Version — © 1999-2001 — QualityMetric, Inc. — All rights reserved

About your local area

This section asks for your views about your **local area**. Think of your local area as everywhere within a ten-minute walk (about half a mile) from your home.

23 How long have you lived in your local area?
If you have lived this area previously and come back again, please just answer about the current period of time that you have lived in your local area.

Write in ☐ years and ☐ months

24 Looking at the faces scale, which face shows best how you feel about living in your local area?

Tick one only

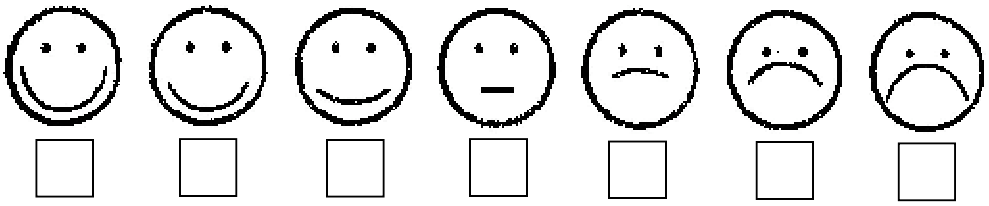

25 For each of the following statements about your local area, please tick one box to show how strongly you agree or disagree.

Tick one per row

**In my local area…**	Strongly agree	Agree	Neither agree nor disagree	Disagree	Strongly disagree
It is pleasant to walk	☐	☐	☐	☐	☐
There is a lot of traffic noise	☐	☐	☐	☐	☐
There is a park within walking distance	☐	☐	☐	☐	☐
The roads are dangerous for cyclists	☐	☐	☐	☐	☐
There is convenient public transport	☐	☐	☐	☐	☐
People are likely to be attacked	☐	☐	☐	☐	☐
There are convenient routes for cycling	☐	☐	☐	☐	☐
There is little green space	☐	☐	☐	☐	☐
It is safe to walk after dark	☐	☐	☐	☐	☐
The nearest shops are too far to walk to	☐	☐	☐	☐	☐
There is little traffic	☐	☐	☐	☐	☐
There are no convenient routes for walking	☐	☐	☐	☐	☐
It is safe to cross the road	☐	☐	☐	☐	☐
The surroundings are unattractive	☐	☐	☐	☐	☐

About your travel

In this section, we are interested in all the journeys you made **yesterday** (between 3 a.m. yesterday morning and 3 a.m. this morning).

26 **Please list each journey you made yesterday to get from place to place.** These might include, for example, going to work, going out to get lunch, coming home from work, going shopping, going to the doctor's, visiting friends, or escorting someone else (e.g. taking a child to school). Please include time spent travelling on foot or by bike, even if this was part of a longer journey (e.g. by bus or train). But please **do not include** journeys you made as part of your job (e.g. as a bus driver), or walking or cycling purely for recreation or exercise (e.g. walking the dog).

We have given you an example of ***one*** *journey. This person walked for ten minutes to the bus stop, rode on the bus for 22 minutes, and then walked for five minutes to get to work (a total of 15 minutes walking). They did not count the time spent waiting for the bus.*

	**What was the purpose of the journey?** Please give a simple description, e.g. "to work", "to get home from work", "shopping", "take child to school"	**How many MINUTES did you spend TRAVELLING by each mode of transport on this journey?** Do not count time spent waiting for buses, trains etc.						
		Bus or coach	Train or underground	Car, taxi or van	Motorcycle or moped	Bicycle	Walking	Other
**Example**	To work	22					15	
**Journey 1**								
**Journey 2**								
**Journey 3**								
**Journey 4**								

Continue over the page if necessary

About your travel (continued)

	What was the purpose of the journey? Please give a simple description, e.g. “to work”, “to get home from work”, “shopping”, “take child to school”	How many MINUTES did you spend TRAVELLING by each mode of transport on this journey? Do not count time spent waiting for buses, trains etc.						
		Bus or coach	Train or underground	Car, taxi or van	Motorcycle or moped	Bicycle	Walking	Other
**Journey 5**								
**Journey 6**								
**Journey 7**								
**Journey 8**								
**Journey 9**								
**Journey 10**								
**Journey 11**								
**Journey 12**								

27 What day of the week was it yesterday? *Write in* [ ] day

28 Were you at home at any time yesterday?

Tick one only Yes [ ] No [ ]

29 Was yesterday a normal working day for you?

Tick one only Yes [ ] No [ ] Not applicable [ ]

About your physical activity

This is the last section of the questionnaire.

In this section, we are interested in finding out about the kinds of physical activities that people do as part of their everyday lives. The questions will ask you about the time you spent being physically active in the **last seven days**. Please answer each question even if you do not consider yourself to be an active person. Please think about the activities you do at work, around your home and garden, to get from place to place, and in your spare time for recreation, exercise or sport.

For each question, write the numbers in the boxes to the left OR tick the box to the right, as appropriate.

30 Think about all the **vigorous** activities that you did in the **last seven days**. Vigorous physical activities refer to activities that take hard physical effort and make you breathe much harder than normal. Think *only* about those physical activities that you did for at least ten minutes at a time.

During the LAST SEVEN DAYS, on how many days did you do vigorous physical activities like heavy lifting, digging, five-a-side football, aerobics, running, or fast cycling?

[ ] days per week ***OR** tick* No vigorous activities [ ] → ***Go to Q. 32***

31 How much time did you spend doing VIGOROUS physical activities on average on each of those days?

[ ] hours and [ ] minutes per day ***OR** tick* Don’t know/not sure [ ]

32 Think about all the **moderate** activities that you did in the **last seven days**. Moderate activities refer to activities that take moderate physical effort and make you breathe somewhat harder than normal. Think *only* about those physical activities that you did for at least ten minutes at a time.

During the LAST SEVEN DAYS, on how many days did you do moderate physical activities like carrying light loads, vacuuming, gardening, dancing, leisurely swimming, or cycling at a regular pace? Do not include walking.

☐ days per week ***OR** tick* No moderate activities ☐ → ***Go to Q. 34***

33 **How much time did you spend doing MODERATE physical activities on average on each of those days?**

☐ hours and ☐ minutes per day ***OR** tick* Don't know/not sure ☐

34 **During the LAST SEVEN DAYS, on how many days did you walk for at least ten minutes at a time?**

☐ days per week ***OR** tick* No walking ☐ → ***Go to Q. 36***

35 **How much time did you spend WALKING on average on each of those days?**

☐ hours and ☐ minutes per day ***OR** tick* Don't know/not sure ☐

36 The last question is about the time you spent **sitting** on weekdays during the **last seven days**. Include time spent at work, at home, while doing course work and during leisure time. This may include time spent sitting at a desk, visiting friends, reading, or sitting or lying down to watch television.

During the LAST SEVEN DAYS, how much time did you spend SITTING on average on each weekday?

☐ hours and ☐ minutes per day ***OR** tick* Don't know/not sure ☐

International Physical Activity Questionnaire — Short last seven days self-administered version (August 2002)

Finally

37 **Please enter today's date.**
We mean the date on which you filled in the questionnaire.

Write in ☐ / ☐ / 05
date *month*

Thank you very much for taking part. Please now return the questionnaire in the envelope provided. No stamp is required. Don't forget to enclose the **signed consent form** if you agree to being contacted again for a follow-up study.
